# Longitudinal *in vivo* bioimaging of hepatocyte transcription factor activity following cholestatic liver injury in mice

**DOI:** 10.1038/srep41874

**Published:** 2017-02-03

**Authors:** Juliette M. K. M. Delhove, Suzanne M. K. Buckley, Dany P. Perocheau, Rajvinder Karda, Patrick Arbuthnot, Neil C. Henderson, Simon N. Waddington, Tristan R. McKay

**Affiliations:** 1 Stem Cell Group, Cardiovascular & Cell Sciences Research Institute, St. George’s University of London, Cranmer Terrace, London SW17 0RE, UK; 2 Gene Transfer Technology Group, Institute for Women’s Health, University College London, 86–96 Chenies Mews, London WC1E 6HX, UK; 3 Wits/SAMRC Antiviral Gene Therapy Research Unit, Faculty of Health Sciences, University of the Witwatersrand, Johannesburg, South Africa; 4 MRC Centre for Inflammation Research, The Queens Medical Research Institute, University of Edinburgh, Edinburgh EH16 4TJ, U.K; 5 School of Healthcare Sciences, Manchester Metropolitan University, Chester Street, Manchester M1 5GD, U.K

## Abstract

Molecular mechanisms regulating liver repair following cholestatic injury remain largely unknown. We have combined a mouse model of acute cholestatic liver injury, partial bile duct ligation (pBDL), with a novel longitudinal bioimaging methodology to quantify transcription factor activity during hepatic injury and repair. We administered lentiviral transcription factor activated luciferase/eGFP reporter (TFAR) cassettes to neonatal mice enabling longitudinal TFAR profiling by continued bioimaging throughout the lives of the animals and following pBDL in adulthood. Neonatal intravascular injection of VSV-G pseudotyped lentivirus resulted in almost exclusive transduction of hepatocytes allowing analysis of hepatocyte-specific transcription factor activity. We recorded acute but transient responses with NF-κB and Smad2/3 TFAR whilst our Notch reporter was repressed over the 40 days of evaluation post-pBDL. The bipotent hepatic progenitor cell line, HepaRG, can be directed to differentiate into hepatocytes and biliary epithelia. We found that forced expression of the Notch inhibitor NUMB in HepaRG resulted in enhanced hepatocyte differentiation and proliferation whereas over-expressing the Notch agonist JAG1 resulted in biliary epithelial differentiation. In conclusion, our data demonstrates that hepatocytes rapidly upregulate NF-κB and Smad2/3 activity, whilst repressing Notch signalling. This transcriptional response to cholestatic liver injury likely promotes partial de-differentiation to allow pro-regenerative proliferation of hepatocytes.

The liver has a unique ability to regenerate. Hepatocytes represent approximately 85% of the liver mass, and are the major metabolic functional units of the liver. Therefore understanding more about the transcriptional regulation of hepatocyte biology, both in health and disease, is key to the rational design of new pro-regenerative therapies. Partial bile duct ligation (pBDL) in mice is a model of acute cholestatic liver injury, which results in increased biliary pressure followed by hepatic injury, inflammation and hepatocyte proliferation^1^. Cholestasis in mice results in the development of a ductular reaction involving neutrophil recruitment, focal zonal necrosis, hepatic progenitor cell (HPC) proliferation and differentiation and hepatic stellate cell (HSC) activation to a matrix-secreting myofibroblast phenotype (Reviewed in ref. 2). The role of resident macrophage Kupffer cells in contentious with depletion experiments in BDL models providing inconclusive data to their role^3^,^4^,^5^. HSC activation is known to be via a TGF-β1 and potentially Notch-dependent mechanism^6^ whilst NF-κB signaling is known to induce hepatocyte proliferation after liver injury^7^. Interestingly, Kim *et al*. have recently shown that NF-κB can itself activate Notch signaling and proliferation in cholangiocytes^8^. CCN1/integrin-activated NF-κB leads to Jagged1/Notch signaling and proliferation of cholangiocytes after BDL. This mechanism also appears to activate HSCs to promote HPC proliferation and differentiation to cholangiocytes. Application of soluble JAG1 after BDL has been shown to reduce hepatic necrosis and ductular reaction^8^. Furthermore, following hepatic injury Wnt3a secretion from macrophages maintains the expression of the Notch inhibitor, Numb, in HPCs promoting their differentiation to hepatocytes^9^. A complex inter-relationship is emerging, which involves intracellular and paracrine signaling, controlling cell mobilization, proliferation and fate decisions in a context-dependent manner during liver development, homeostasis and injury.

We have developed an *in vivo* platform for quantifying hepatocyte transcription factor activity in live mice. We used lentiviral transcription factor activated reporter cassettes (TFAR) co-expressing firefly luciferase and eGFP. Conditional control of reporter gene activity by a synthetic promoter enabled longitudinal analysis of transcription factor activity profiles by continued bioimaging in mice following pBDL. Intravascular injection of VSV-G pseudotyped lentiviruses into newborn mice resulted in almost exclusive transduction of hepatocytes, which allowed analysis of hepatocyte-specific transcription factor activity. This has enabled us to longitudinally assess hepatocyte NF-κB, Smad 2/3, Notch and Wnt signaling activity *in vivo* following pBDL. We then combined TFAR bioimaging with complementary immunohistochemistry and *in vitro* evaluation of hepatic progenitor cell (HepaRG cells) differentiation to provide new insights into the temporal transcriptional activity and signaling mechanisms that mediate the hepatic injury and repair response during cholestatic liver injury.

## Results

### Lentiviral transduction is restricted largely to hepatocytes after intravenous administration to neonatal mice

**Figure 1:**
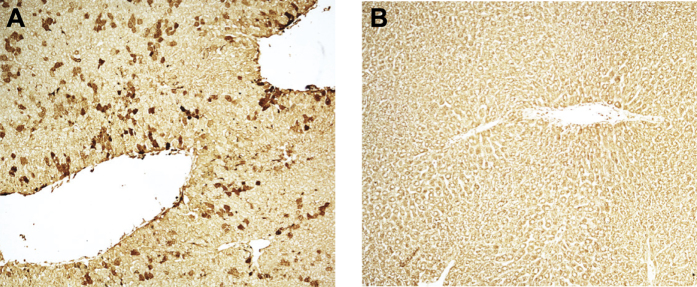
Neonatal intravascular administration of lentiviral vectors targets the liver and overwhelmingly transduces hepatocytes. Lentivirus constitutively expressing FLuc and GFP (LNT-SFFV-FLuc/eGFP) was administered to P1 neonatal mice by intravascular injection. Mice were sacrificed after 90 days and liver sections immunostained with GFP antibody. (**A**) GFP expression was overwhelmingly evident in hepatocytes compared to (**B**) controls (n = 3 both groups).

**Figure 2:**
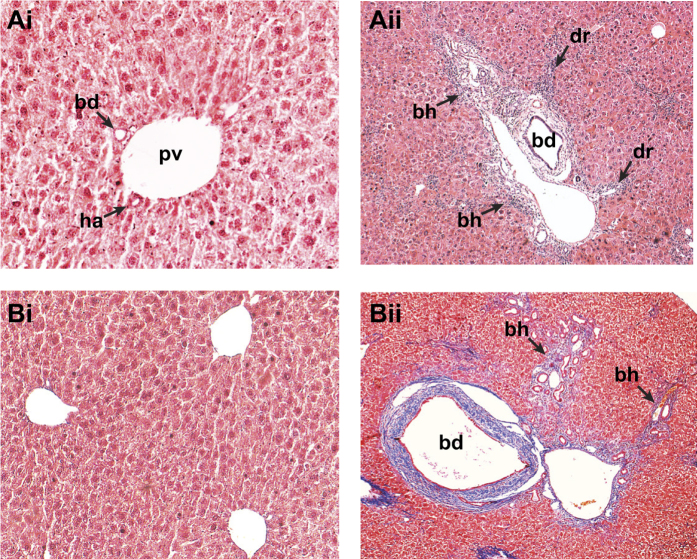
Partial bile duct ligation results in liver remodelling and collagen deposition in occluded lobes. Adult mice were subjected to partial bile duct ligation and sacrificed after 90 days. Control livers from sham operated mice (**Ai**) and occluded pBDL livers (**Aii**) were fixed, sectioned and stained with Hematoxylin & Eosin for histological architecture and Lilly’s trichrome (**Bi** & **ii**) staining collagen (blue), nuclei (black) and cytoplasm (red). Bile duct (bd), portal vein (pv), biliary hyperplasia (bh), ductular reaction (dr), (n = 3 both groups).

Here we report, for the first time, that intravenous administration of VSV-G pseudotyped lentivirus constitutively expressing FLuc/eGFP in P0 mouse neonates results in efficient liver-restricted hepatocyte transduction (Fig. 1A and B) that is maintained for over 100 days^10^. Bile duct ligation is an established model for initiation of the ductular reaction. However, complete occlusion of the common bile duct in mice can result in severe hepatic injury, liver failure and mortality. Partial bile duct ligation (pBDL) can be achieved by either a partial occlusion of the common bile duct^11^ or a complete occlusion of the ducts draining the median and left liver lobes leaving the right and caudate lobes fully functional^12^ and therefore we used pBDL throughout this study. Following pBDL, we observed bile duct dilatation, biliary hyperplasia, and mononuclear cell infiltrate characteristic of portal tract ductular reaction (Fig. 2Ai and ii). Furthermore, an increase in collagen deposition was observed following pBDL (Fig. 2Bi and ii).

**Figure 3:**
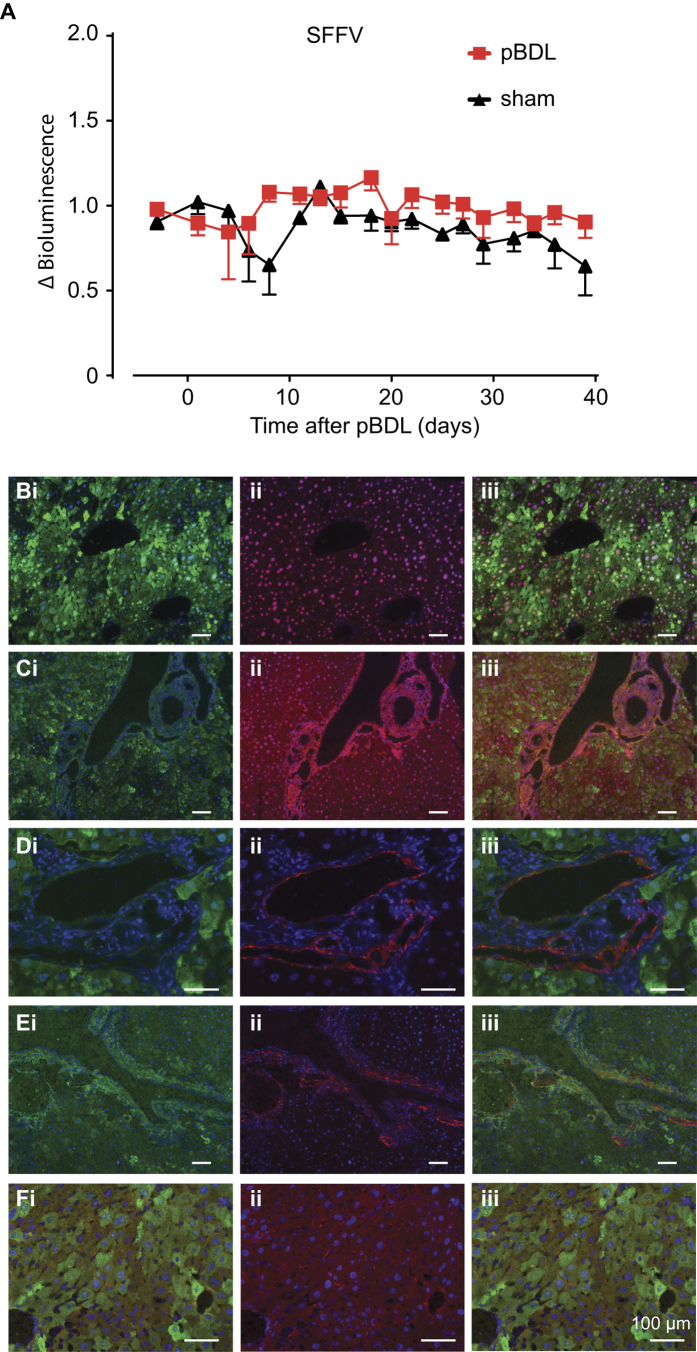
Reporter gene expression is stable and restricted to hepatocytes after pBDL following neonatal intravascular administration of lentiviral vectors. SFFV-FLuc/eGFP lentivirus was administered to P1 neonatal mice by intravascular (i.v.) injection and then subjected to pBDL or sham pBDL in adulthood. (**A**) Mice were subjected to continued luciferase bioimaging over 40 days where no change in luciferase activity was observed over time or between pBDL and sham groups (n = 10 pBDL, 5 sham, not significant, Student’s t-test). Mice were sacrificed 90 days after pBDL and co-immunostained for GFP and markers of (**Bi-iii**) hepatocytes; HNF4α, (**C-iii**) biliary epithelia; CK7, (**Di-iii**) hepatic progenitors; PKM2, (**Ei-iii**) myofibroblasts; αSMA and (**Fi-iii**) hepatic stellate cells; GFAP (all groups n = 3–6).

We initially administered a VSV-G pseudotyped lentivirus constitutively expressing FLuc/eGFP from the SFFV promoter to neonatal mice, which upon maturity, were subjected to pBDL or sham surgery followed by continued luciferase bioimaging. There was no difference in luciferase activity between pBDL and sham-treated animals. Similarly there was no change in reporter activity for up to 40 days post pBDL (Fig. 3A). Mice were sacrificed 90 days later to correlate lentiviral transduction with liver pathology at the cellular level. Intravenous administration of lentivirus resulted in up to 49% GFP positive hepatocytes. The vast majority of cells stably expressing eGFP 90 days post-pBDL were HNF4α^+^ hepatocytes (Fig. 3Bi–iii) Areas of ductular reaction were distinctly lacking in GFP expression. Furthermore GFP expression did not co-localise with markers of cholangiocytes (CK7, Fig. 3Ci–iii), hepatic progenitor cells (HPC – PKM2, Fig. 3Di–iii), myofibroblasts (α-SMA, Fig. 3Ei–iii) or quiescent hepatic stellate cells (HSC – GFAP, Fig. 3Fi–iii).

### Hepatocyte specific TFAR activity after pBDL in adult mice

**Figure 4:**
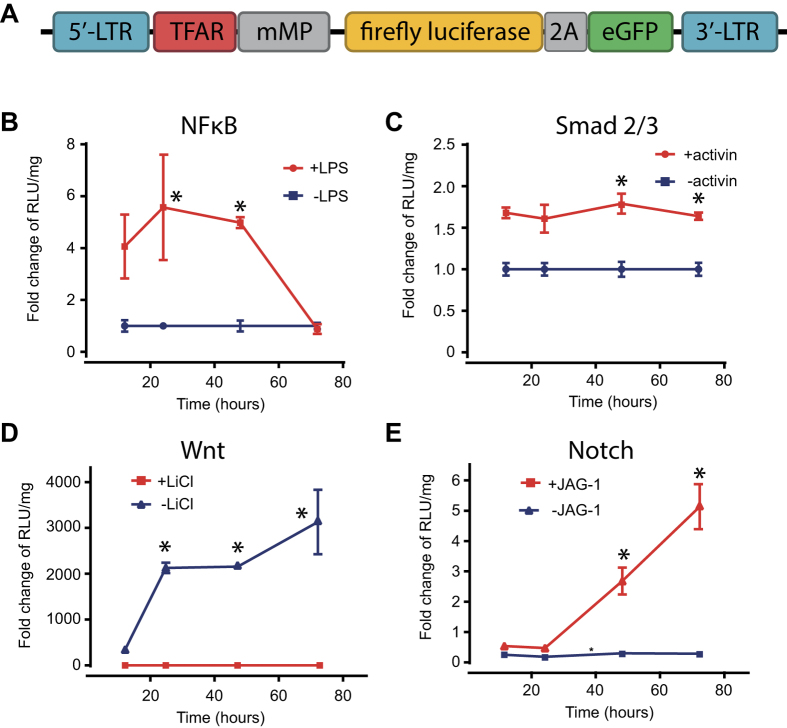
*In vitro* validation of transcription factor activated lentiviral reporter vectors. (**A**) Schematic representation of parental lentiviral reporter construct. (**B–E**) 293 T cells were transduced with VSV-G pseudotyped TFAR lentiviral vectors at an MOI of 10 then incubated with the relevant agonists and luciferase expression quantified at 12, 24, 48 and 72 h (n = 3–6, mean+/− SEM, comparison by analysis of variance with repeated measures).

In this study we used our previously described liver-targeted transcription factor activated reporter (TFAR) cassettes^10^ to investigate hepatocyte-specific signalling during acute cholestatic liver injury. We have previously generated a library of lentiviral TFAR where serial transcription factor consensus sites are engineered upstream of a minimal promoter sequence driving a bicistronic firefly luciferase (FLuc)/eGFP reporter cassette (Fig. 4A). For this study we employed TFARs that were specific for four cell signalling pathways implicated in responses to cholestatic liver injury: NF-κB, TGF-β (Smad2/3), Wnt (TCF/LEF) and Notch. We initially validated each TFAR for activation over 72 h in HEK293T cells. NF-κB, TGF-β and Wnt were all responsive to their respective agonists (LPS, Activin A and LiCl2 respectively) (Fig. 4B–D) and Notch TFAR transduced cells were responsive to co-culture with JAG1 over-expressing 293 T cells in a dose dependent manner (Fig. 4E).

**Figure 5:**
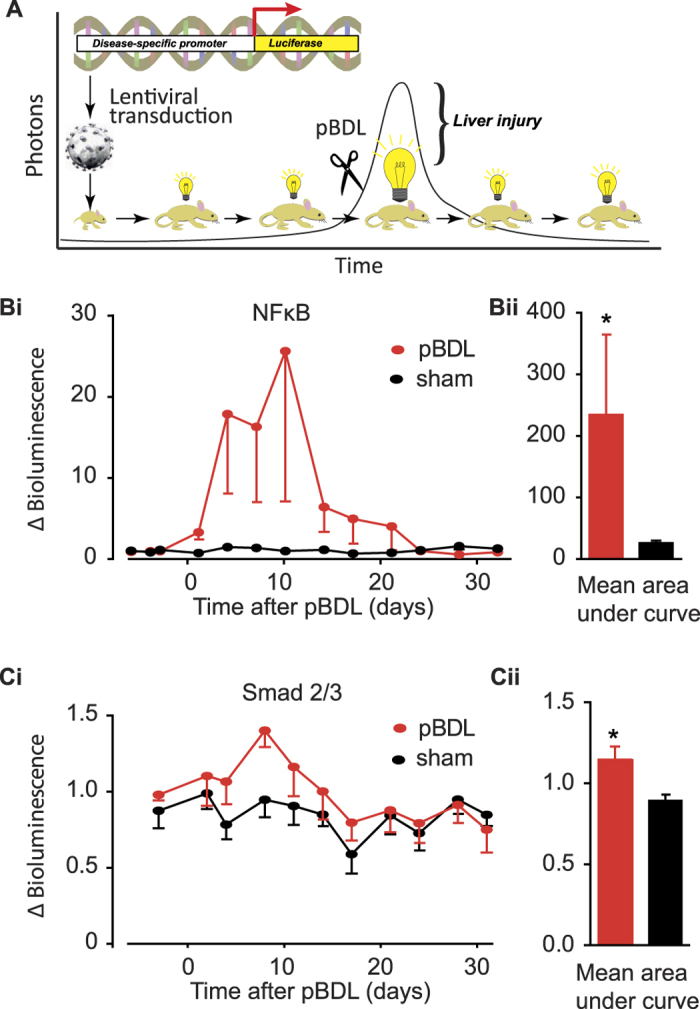
pBDL causes transient activation of NFB and Smad2/3 transcription factor activity. (**A**) Cartoon of the principles underlying somatotransgenic bioimaging. Lentiviruses expressing transcription factor activated reporter (TFAR) constructs expressing luciferase and GFP are administered to neonates. When they reach adulthood baseline luciferase expression is assessed before inducing a disease state, in this case by pBDL. Continued luciferase bioimaging provides a quantifiable surrogate for nuclear localization and target DNA binding of transcription factors. Somatotransgenic mice were generated as described then subjected to pBDL or sham pBDL and luciferase activity assayed over 30 days. (**Bi** & **ii**) NFκB and (**Ci** & **ii**) Smad2/3 TFAR activity showed significant increases as assessed by area under the curve analysis (NFκB, n = 13 pBDL, n = 6 sham; p < 0.0001; Smad2/3, n = 9 pBDL, n = 9 sham, p < 0.05, Student’s t-test).

*In vivo* transcription factor activity analysis was performed by neonatal administration of lentivirus containing the biosensing TFAR. Signalling pathway activation during disease induction was then quantified as photonic output (schematically represented in Fig. 5A). Having shown that lentiviral transduction is almost exclusively hepatocyte-specific and maintained in daughter cells because of stable genomic integration of the expression cassette, we can assume that the expansion of FLuc/eGFP expression we observe during development is largely from hepatocytes. Our data generated using a constitutively activated GFP also suggest that following pBDL, most of the resident hepatocyte population survives and remains stable during liver remodelling post pBDL.

We next carried out longitudinal luciferase bioimaging in mice containing each of the four TFAR (NF-κB, TGF-β (Smad2/3), Wnt (TCF/LEF) and Notch) following pBDL at 49–60 days post-birth. We observed an acute NF-κB response immediately after pBDL which was not apparent in sham operated controls (Fig. 5Bi and ii). Control mice were subjected to laparotomy to correct for any paracrine inflammatory response consequent to the abdominal surgery. Interestingly, in a number of the sham treated mice an NF-κB response in hepatocytes above baseline was observed that quickly diminished (Supplementary Fig. 1). This would be consistent with a systemic inflammatory response to the laparotomy. The NF-κB response peaked 10 days after pBDL and decreased to baseline by 24 days. This was revealed by a more than 28-fold increase in NF-κB activity following pBDL when compared to controls, as revealed by analysis of the area under curve. A similar statistically significant, but less pronounced, response was seen with the TGF-β activated Smad2/3 TFAR at the same timepoints (Fig. 5Ci and ii). Collectively, these data demonstrate that hepatocytes are important “primary responders” to inflammatory and fibrotic stimuli following biliary injury.

**Figure 6:**
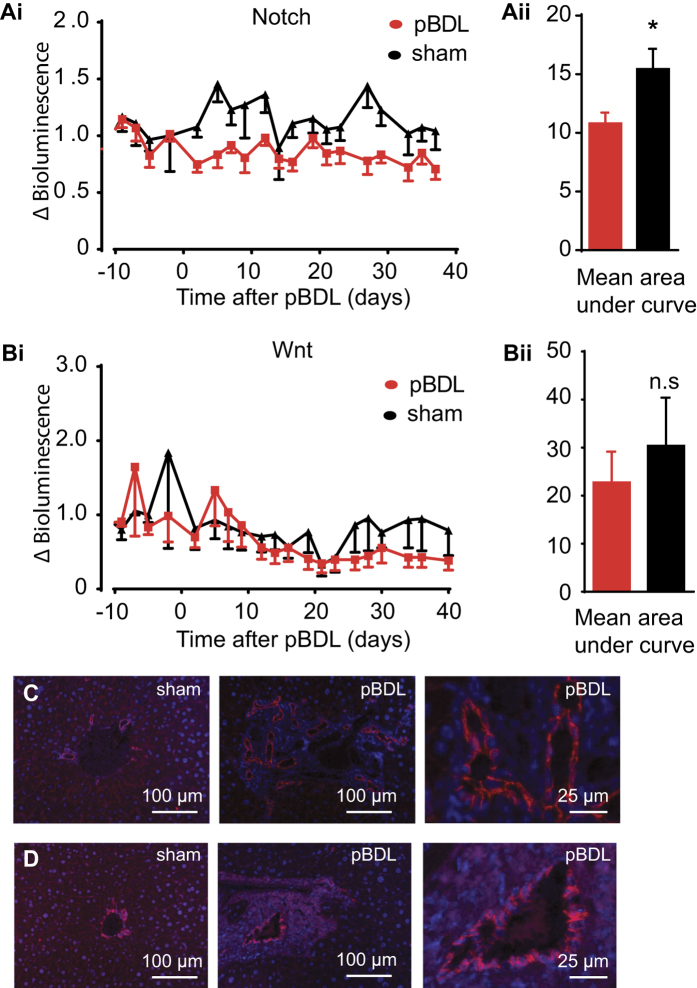
pBDL causes repression of Notch signalling. Mice somatotransgenic for liver specific expression of the Notch and Wnt (TCF/LEF) TFAR were subjected to pBDL or sham surgery. There was significant repression of the (**Ai** & **ii**) Notch TFAR over 40 days pBDL compared to sham control analysed by area under the curve comparison (n = 10 pBDL, n = 5 sham; p < 0.05, Student’s t-test) but no significant change in (**Bi** & **ii**) Wnt TFAR activity (n = 8 pBDL, n = 5 sham, not significant, Student’s t-test). Immunohistochemistry of liver sections 90 days after pBDL showed an increase in the number of (**C**) NICD positive and (**D**) β-Catenin positive bile ducts in pBDL compared to sham (n = 3).

The Notch and Wnt pathways have been implicated in regeneration and repair after hepatic injury^9^. To analyse the role of these pathways following pBDL, adult liver targeted TFAR mice were assessed by continued bioimaging as before. The Notch TFAR mice showed a prolonged and significant decrease in luciferase reporter activity in pBDL compared to sham-treated animals (Fig. 6Ai and ii) but the Wnt activated TCF/LEF TFAR showed no significant change in activity (Fig. 6Bi and ii). Histological assessment of activated NICD and β-catenin showed largely membrane-bound localization in cholangiocytes in sham and pBDL-treated mice (Fig. 6C and D). Collectively, these data indicate that hepatocyte Notch transcription factor activity is decreased following pBDL, and notch signalling is localized to the bile ducts in normal and hyperplastic livers after pBDL. It is possible that hepatocytes inhibit Notch signals that are emanating from hyperplastic bile ducts in pBDL livers.

### Interrogation of Wnt and Notch signalling in differentiated HepaRG cultures

**Figure 7:**
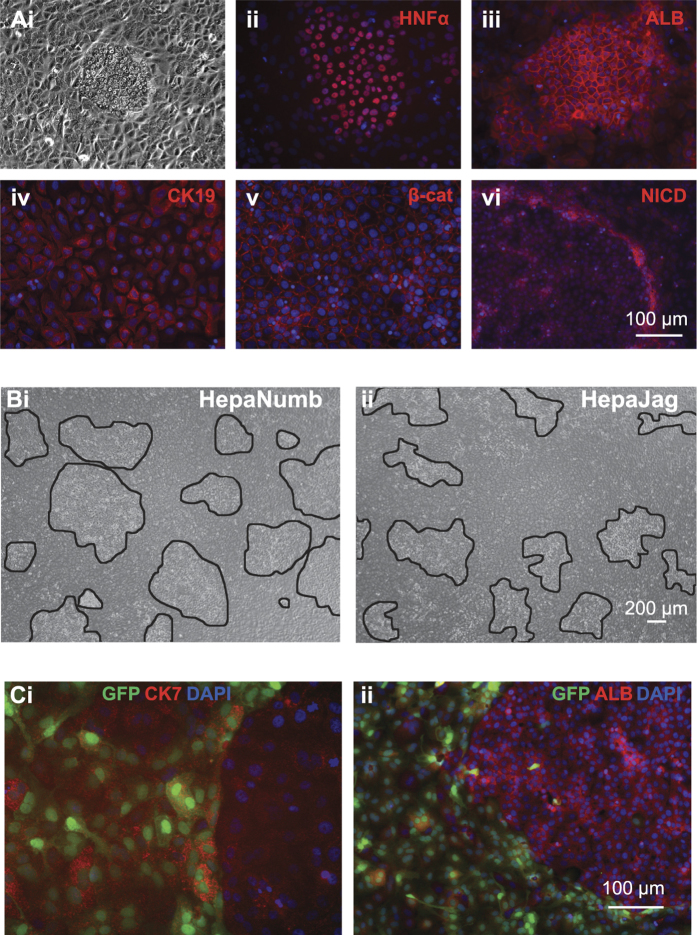
Modulation of Notch signalling influences the differentiation of bipotent HepaRG cells. HepaRG cells subjected to differentiation conditions formed (**Ai**) tight colonies that immunostained positive for the hepatocyte markers (**Aii**) HNF4α and (**Aiii**) Albumin whereas the surrounding cells immunostained positive for the biliary epithelial marker (**Aiv**) CK19 and (**Av**) β-Catenin. The interface between colonies and surrounding cells immunostained positively for (**Avi**) NICD. Bipotent HepaRG cells were transduced with lentiviral constructs expressing Numb or Jag1/GFP then subjected to differentiation conditions. We observed a greater area of hepatocyte-like colonies in (**Bi**) HepaRG-Numb cells compared to (**Bii**) HepaRG-Jag1/GFP cells as quantified by total area ImageJ software. In HepaRG-Jag1/GFP cultures dual immunocytochemistry revealed that (**Ci**) GFP and CK7 co-localized but (**Cii**) GFP and Albumin never co-localized.

To interrogate the interactions of hepatocytes and cholangiocytes we exploited the bipotent nature of the HepaRG cell line. Bipotent HepaRG progenitors were subjected to a differentiation protocol previously described by Dianat *et al*.^13^. The protocol was developed to differentiate HepaRG to cholangiocytes but in our hands it reproducibly generated CK19^+^ cholangiocyte cultures interspersed with colonies of HNF4α^+^/ALB^+^ hepatocytes (Fig. 7Ai–iv). In agreement with our evaluations *in vivo*, we observed widespread membrane-bound β-catenin staining in CK19-positive cholangiocytes and interestingly, increased NICD staining at the interface between cholangiocytes and the hepatocyte colonies (Fig. 7Av and vi). We compared the effects of modulating Notch signalling by transducing HepaRG cells with lentiviruses constitutively expressing the Notch inhibitor NUMB (HepaNumb) or the Notch ligand JAG1 (HepaJag) before subjecting them to our differentiation protocol. There was a significantly higher total area of hepatocyte-like colonies in HepNumb cultures (36.94%, n = 14) compared to HepaJag (22.05%, n = 12) (Fig. 7Bi and ii). The transgenes were expressed from a bicistronic lentiviral cassette co-expressing GFP from a truncated CMV promoter and transduction efficiencies ranged between 50 and 80%. Interestingly, we did not observe Jag1/GFP expression in any hepatocyte-like colonies (Fig. 7Ci and ii). We imply from these data that hepatocyte differentiation can only occur in HepaRG cells when Notch activity is repressed. This is consistent with increased hepatocyte colony formation in Numb transduced HepaRG cultures.

## Discussion

Bile duct ligation in rodents results in acute cholestasis and a ductular reaction with upregulation of inflammatory and pro-fibrotic signalling^14^. Complete ligation of the common bile duct causes high rates of mortality but partial bile duct ligation (pBDL), occluding the ducts draining the left and median lobes, results in localized cholestasis that is amenable to longer-term analysis^15^. NF-κB and TGF-β1 signaling are prominent in promoting responses to liver injury such as myofibroblastic differentiation and ECM remodelling^16^,^17^. Although the role of TGF-β signaling is well characterised in HSC, activity in hepatocytes after liver injury is less clear. Two previous studies have indicated that following liver injury TGF-β signalling emanates from hepatocytes but neither present a longitudinal analysis^17^,^18^. We sought to investigate longitudinal transcription factor activity *in vivo* using bioluminescence imaging in live mice subjected to pBDL. A constitutively activated eGFP reporter was used to assess the distribution of lentiviral transduction > 90 days post-transduction. In line with our previous observations, transduction was largely restricted to the liver but, unexpectedly, histological analysis revealed that transduced cells within the liver were almost exclusively hepatocytes. Specifically, we observed no evidence of cholangiocyte, HSC or HPC transduction pre- or post-BDL inferring that we were not transducing bi-potent progenitor cells. This provided us with a unique tool to assess transcription factor activity specifically in hepatocytes subsequent to pBDL.

Bile duct ligation results in an acute phase inflammatory response leading to necrosis^19^ caused directly by bile acid reflux^20^ and other deregulated factors such as osteopontin^21^. There is strong evidence from mice that NF-κB activation during this inflammatory phase promotes hepatocyte proliferation *in vivo* following partial hepatectomy^7^,^22^. In hepatocytes we saw an acute increase in both NF-κB and Smad2/3-mediated luciferase activity after pBDL, peaking at around 10 days post-BDL before returning to pre-pBDL levels at 28 days. Interestingly, NF-κB and Smad2/3 responses returned to pre-pBDL levels within the same timeframe, whereas it is known that liver fibrosis in partial BDL models progresses over many months^23^. This would suggest that compensatory factors restore a homeostatic balance to reduce damage and maintain function despite chronic disease. This would be consistent, for example, with Smad7 upregulation repressing Smad2/3 responses despite continued expression of TGF-β1^17^. Although the role of TGF-β signalling in hepatocytes is not clearly understood it is well established that TGF-β promotes the ductular reaction in BDL rats by promoting cholangiocyte proliferation in an αvβ6-integrin specific manner^24^,^25^. Our data is the first hepatocyte-targeted description of NF-κB and Smad2/3 activated luciferase reporter bioimaging in a rodent pre-clinical model of acute cholestatic liver injury.

Notch signalling is established as playing a significant role in bile duct development where the Notch ligand JAG1 promotes cholangiocyte differentiation of bi-potent HPCs^26^. NF-κB and Notch signalling have been linked during HPC commitment to cholangiocytes during the ductular reaction in mice^8^, and also to the severity of human hepatic fibrosis^6^ but never related specifically in hepatocytes after pBDL. Haploinsufficiency at the locus encoding the Notch ligand *JAG1* results in Alagille syndrome characterised by impaired intrahepatic bile duct development and widespread ductular reaction. There is no obvious effect on hepatocytes^27^ and Notch inhibition by Wnt3a-mediated Numb upregulation in HPCs results in hepatocyte specification^9^. Our longitudinal assessment indicated that Notch signalling is actually inhibited in hepatocytes over 50 days post-pBDL whilst there is no change in Wnt-mediated TCF/LEF activity throughout a similar timecourse. Immunohistochemistry showed that both β-catenin and NICD present in cholangiocytes but largely absent from hepatocytes. The absence of NICD in hepatocytes is consistent with upstream inhibition of Notch receptor cleavage as protection from perpetuating Notch signalling emanating from the ductular reaction.

Transduction of the bi-potent hepatic progenitor cell line HepaRG with lentiviruses constitutively expressing Numb or JAG1 further corroborates this hypothesis. Numb over-expression resulted in an increase in the frequency and size of ALB^+^/HNF4α^+^ clusters seen within differentiated cultures compared to controls, indicating that reduced Notch signalling confers a propensity for cells to differentiate towards the hepatocyte lineage. Differentiated HepaRG cultures over-expressing JAG1 had less hepatocyte-like colonies. Interestingly, bi-potent HepaRG transduced with the JAG1-GFP expressing lentivirus were unable to differentiate to hepatocyte colonies. It is possible that this observation is a result of differential methylation of the CMV promoter in hepatocytes compared to cholangiocytes, or that hepatocyte differentiation is not possible in the presence of JAG1. With either situation hepatocyte-like colonies do not have exogenous JAG1 expression. There is some evidence that Notch inhibition promotes hepatocyte proliferation and in some cases metastases^28^,^29^ implying hepatocytes are intrinsically resistant to Notch signalling^30^. The emerging consensus is that regenerative hepatocyte proliferation after acute liver injury does not emanate from progenitors residing within the canal of Hering^31^. Overall, our novel data highlights the use of longitudinal luciferase bioimaging in monitoring cell-type specific transcriptional activity in live animals. This has enhanced our understanding of the temporal activity profiles of the core transcriptional networks involved during acute cholestatic liver injury and repair.

## Methods

### Construction and production lentiviral reporter vectors

The NF-κB, SMAD2/3, Notch and Wnt (TCF/LEF) TFAR lentiviral vector cassettes were previously described in Buckley *et al*.^10^.

HEK293T viral producer cells were seeded at 2 × 10^7^ cells per T175 cm^2^ flask and transfected using 50 μg pLNT-TFBE-JDG, 17.5 μg pMD2.G (VSV-G envelope plasmid), and 32.5 μg pCMVΔR8.74 (gag-pol packaging plasmid) pre-complexed with 1 μl polyethylenimine (10 mM) (Sigma-Aldrich) in OptiMEM for 3 h. Transfection media was replaced with complete DMEM and viral supernatant collected at 48 and 72 hours, filter sterilized (0.45 μM), and concentrated by overnight low-g centrifugation. Pellets were resuspended in OptiMEM and stored at −80 °C. All viruses were titered using a p24 assay (Zeptometrix, Buffalo, NY, USA) as per manufacturer’s instructions.

### *In vitro* validation of reporter gene activity

HEK293T, HeLa and NIH3T3 cells were cultured in DMEM supplemented with 10% FBS, 1% penicillin/streptomycin (Sigma), 2mM L-glutamine and 1x non-essential amino acids. HepaRG cells were cultured in William’s E medium containing Glutamax and sodium pyruvate supplemented with 10% FBS, 1% Pen/Strep, 1x non-essential amino acids, 4 μg/ml human recombinant insulin zinc solution, and 50 μM hydrocortisone. All reagents were sourced from Life Technologies.

For the *in vitro* validation of NF-κB, SMAD2/3 and Wnt (TCF/LEF) TFAR, cells were transduced with VSV-G pseudotyped lentivirus reporters at an MOI of 10. Small molecule or growth factor agonists or inhibitors were used for reporter functional validation. The trophoblast-isolated SGHPL5 cell line was transduced with a Notch reporter lentivirus to validate this vector. Varying seeding densities of *JAGGED-1*-overexpressing HEK293T cells were used to activate the Notch-SGHPL5 cells. Analysis was performed following 72 hours of co-culture.

Gene activation was quantified by luciferase assay on cell lysates as follows: 20 μl assay buffer (25 mM Tris phosphate pH 7.8, 1 mM DTT, 1 mM EDTA, 1% Triton X-100, 8 mM MgCl_2_, 3 ml glycerol, 1.25 mM rATP, 0.5% BSA) was added to 20 μl cell extract. Luciferin (Gold Biotechnology, MO, USA) was added to a final concentration of 1.5 mM and luminescence measured using the POLARstar Omega microplate reader (BMG Labtech). Analysis performed using MARS data analysis software (BMG Labtech). Relative light units were normalized relative to total protein as determined by Bradford assay (BioRad) using manufacturer’s instructions.

### Differentiation of HepaRG cells

HepaRG cells were seeded at low density and maintained in William’s E medium containing 10% FBS, 1x non-essential amino acids, hydrocortisone (50 μM) and insulin (4 μg/ml). Cells were plated onto collagen-coated plates and allowed to grow to confluency prior to chemical induction. Confluent cells were treated with IL-6 (10 ng/ml) (R&D Systems) for 2 days, followed by 2 days of sodium taurocholate hydrate (10 nM) (Sigma) treatment, and a further 8 days of sodium taurocholate hydrate (10 nM) (Sigma) and sodium butyrate (1.8 μM) (Sigma) treatment.

### Immunocytochemistry

Media was removed and cells washed 3x in PBS. Cells were fixed in 4% paraformaldehyde (PFA) (Sigma) for 20 minutes followed by three washes in PBS. Cells were incubated in blocking buffer (PBS, 0.3% Triton X-100, 1% BSA) for one hour at room temperature before the addition of primary antibody (Table 1) resuspended in blocking buffer was added to the cells and incubated overnight at 4 °C. Cells were washed three times in PBS for 5 minutes followed by incubation at room temperature for 1 hour with fluorophore-conjugated secondary antibody resuspended in blocking buffer. Cells were washed 3x in PBS and incubated in 1 × 4′,6-diamidino-2-phenylindole (DAPI)(Sigma) in PBS for 5 minutes before being washed 3x in PBS for 5 minutes. Cell F imaging software was used for all analysis.

### Animal procedures

Unless otherwise stated, outbred CD1 mice (Charles River) were time mated to produce neonatal animals. At postnatal day 1, neonates were briefly anesthetized (on ice) and lentivirus injected by intravenous (via the superficial temporal vein) injection.

All experiments were performed in accordance with relevant guideless and regulations: Experiments were carried out under United Kingdom Home Office regulations and approved by the ethical review committees of Imperial College London and University College London.

### Partial bile duct ligation in mice

At 49 (SFFV), 50 (NF-κB, Wnt, Notch), or 60 (Smad 2/3) days post-birth, mice were anaesthetized with isoflurane (Abbott Laboratories) and a midline laparotomy (≈15 mm) performed just caudal to the sternum. The median and left lobes of the liver were exteriorized and kept moist with sterile gauze. The bile duct was ligated with 6–0 silk suture to occlude outflow from the left and median lobes but not occluding bile outflow from the right and caudate lobes as described by Yang and colleagues^32^. The liver was returned to the abdomen and the wound closed in two stages using 6–0 silk suture; skin was closed using a subcuticular suture with buried knots. Sham operated mice received laparotomy only. Mice were allowed to recover in a warmed chamber. Pre- and post-operative buprenorphine analgesia was provided.

### Bioluminescence imaging

Unless otherwise stated, mice were anaesthetized with isoflurane (Abbott Laboratories), injected i.p. with firefly D-luciferin (15 mg/ml in PBS; Gold Biotechnology) and imaged 5 min later with a cooled charge-coupled device (CCCD) camera (IVIS; PerkinElmer). Luciferin dose was 150 mg/kg; volume varied with age/size of animal, for example, adult mice were given 300μl. Grey-scale photographs were acquired with a 24-cm field of view and then a bioluminescence image was obtained using a binning (resolution) factor of 4, a 1.2/f stop and open filter. Regions of interest (ROIs) were defined manually using a standard area for each organ under investigation. Signal intensities were calculated with Living Image software (Perkin Elmer) and expressed as photons per second per cm^2^ per steradian. Where possible, BLI was carried out in adult reporter rodents on three consecutive days to establish a robust median baseline; subsequent data points were expressed as fold-change over this internal standard for each individual animal.

### Immunohistochemistry

Liver tissue was fixed in 10% formalin overnight, dehydrated using 70%, 80%, 90% and 2 × 100% ethanol for 1 hour each followed by Histoclear (National Diagnostics) overnight. Samples were embedded in Fibrowax (VWR). Tissue sections were cleared twice in Histo-clear for 10 minutes each, and brought to water using 90%, 70%, and 50% IMS, followed by 5 minutes in distilled water. Antigen retrieval was performed in 10 mM citrate buffer (pH6.0)(Sigma-Aldrich) or 10 mM Tris HCL for 40 or 10 minutes respectively and cooled for 20 minutes. Samples were incubated for 10 minutes in TBS containing 0.3% Triton X-100 (TBS-T) followed by blocking in TBS-T + 15% normal goat serum (NGS) (Vector laboratories) for 1 hour at room temperature. Primary antibody (see Table 2) was diluted in TBS-T/10% NGS overnight at 4 °C. Slides were rinsed thrice for 5 minute in TBS followed by incubation in secondary antibody diluted in TBS-T/10% NGS for 1 hour at room temperature. Samples were washed thrice for 5 minutes in TBS before incubation with 0.3% Sudan Black B made up in 70% ethanol for 15 minutes at room temperature. Sections were rinsed thrice with TBS for 5 minutes and mounted using Fluorescence Mounting Medium (Dako). Images in several fields were quantified using ImageJ software as outlined by Wang *et al*.^33^. Images were subjected to thresholding to determine true GFP expression over background with pixels between background level (79) and the maximum (255) considered positive. Positive pixels as a percentage of total image pixels were used to determine total area of GFP positive regions.

### H&E and Lilley’s trichrome staining

Histological sections were brought to water as previously described before being placed in Weigert’s haematoxylin for 5 minutes and then rinsed in running water. Differentiation, or selective stain removal was performed by incubating the slides in acid alcohol (70% EtOH with 0.5% HCl) for approximately 10. Sections were rinsed in water and subsequently stained in eosin for 5 minutes. This was followed by rapid dehydration by passing through 70%, 90% and 100% alcohol for 2–3 seconds followed by a final 100% alcohol bath for 5 minutes. Incubation in 2 baths of Histo-clear for 10 minutes each was performed dry before mounting with Histomount (Sigma).

### Lilley’s trichrome staining

Histological sections were brought to water as in protocol above, incubated in Weigert’s haematoxylin for 5 minutes and rinsed in distilled water. Slides were placed in working strength picric acid solution for 10 minutes, washed in distilled water, and stained in Xylidine Red solution (Sigma) for 1 minute and rinsed in distilled water. Differentiation was performed using a 1% phosphomolybdic acid bath for 10 minutes. Collagen staining was achieved by subsequently staining slides in Aniline Blue solution (Sigma) for 1 minute before being placed in fresh 1% phosphomolybdic acid for 2 minutes and 1% acetic acid for 3 minutes prior to rapid dehydration of samples in 95% and 2 × 100% alcohol. Slides were cleared in 2 baths of Histoclear for 10 minutes each before mounting with Histomount.

### Statistical analysis

Statistical analysis on *in vitro* vector analysis data was performed using an unpaired Student’s one-tailed t-test. All data are expressed as mean values ± SEM, with each sample being measured at least in triplicate. For *in vivo* data, repeated measurements were analyzed with repeated measures analysis of variance with significance level of p < 0.05. Area under curve data for experiments with two groups was analyzed by Student’s one-tailed t-test. For areas under curve derived from two or more groups, one-way analysis of variance with Newman-Keuls post-hoc multiple comparison test was performed.

## Additional Information

**How to cite this article**: Delhove, J. M. K. M. *et al*. Longitudinal *in vivo* bioimaging of hepatocyte transcription factor activity following cholestatic liver injury in mice. *Sci. Rep.* **7**, 41874; doi: 10.1038/srep41874 (2017).

**Publisher's note:** Springer Nature remains neutral with regard to jurisdictional claims in published maps and institutional affiliations.

## Supplementary Material

Supplementary Figure 1

## Figures and Tables

**Table 1:** Primary and secondary antibody information used for immunocytochemistry.

Antibody	Supplier	Dilution
Mouse αCK19	Dako (M0888)	1:200
Mouse αCK7	Sigma (4465 P)	1:200
Mouse αHNF-4α	Perseus (PP-H1415–00)	1:200
Mouse αALB	R&D Systems (MAB1455)	1:200
Goat αSox9	R&D Systems (AF3075)	1:500
Rabbit αNICD	Abcam (ab8925)	1:200
Rabbit (active) β-catenin	New England Biolabs (8814 S)	1:200
Rabbit α-goat Alexafluor	Life Technologies (A11079)	1:500
Goat α-rabbit Alexafluor	Life Technologies (A11011)	1:500
Goat α-mouse Alexafluor	Life Technologies (A11031)	1:500
DAPI	Sigma Adlrich (D9542)	1:1000

**Table 2:** Primary and secondary antibodies used for immunohistochemistry.

Antibody	Supplier	Antigen Retrieval	Dilution
Chicken αGFP	NEB (13970)	Na Citrate (pH6)	1:300
Mouse αCK7	Perseus (PP-H415-00)	Na Citrate (pH6)	1:200
Mouse αHNF-4α	Dako (M0851)	Tris HCl (pH10)	1:100
Rabbit αSMA	Dako (Z0334)	Na Citrate (pH6)	1:200
Rabbit αGFAP	Dako (M7018)	Tris HCl (pH10)	1:500
Rabbit α-PKM2	Life Technologies (PA5-23034)	Na Citrate (pH6)	1:200
Goat α-rabbit Alexafluor	Life Technologies (A-11011)		1:500
Goat α-mouse Alexafluor	Life Technologies (A-11031)		1:500
Goat α-chicken Alexafluor	R&D Systems (NL018)		1:400
DAPI	Sigma Aldrich (D9542)		1:1000
