# Supplementary material for: Longitudinal in vivo bioimaging of hepatocyte transcription factor activity following cholestatic liver injury in mice
Source: Sci Rep. 2017 Feb 3;7:41874. doi: 10.1038/srep41874 (PMC5291111; doi:10.1038/srep41874)
Supplement: Supplementary Figure 1 [file srep41874-s1.pdf]

Longitudinal *in vivo* bioimaging of hepatocyte transcription factor activity following cholestatic liver injury in mice

Juliette MKM Delhove, Suzanne MK Buckley, Dany P Perocheau, Rajvinder Karda, Patrick Arbuthnot, Neil C Henderson, Simon N Waddington & Tristan R McKay

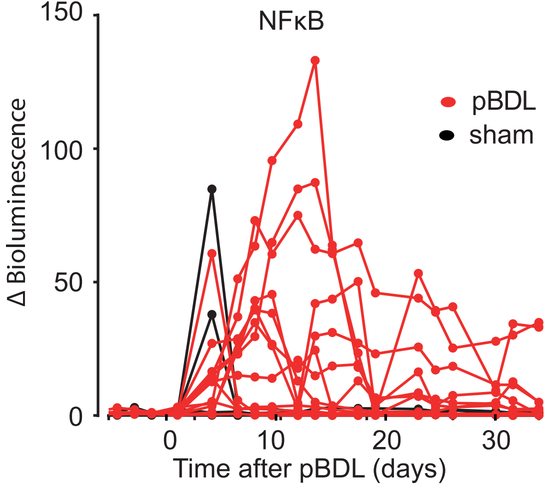
NFκB
Δ Bioluminescence
150
100
50
0
0
10
20
30
Time after pBDL (days)
pBDL
sham
